# Two Hundred Cases of Tonsilitis

**Published:** 1885-12

**Authors:** J. M. G. Carter

**Affiliations:** President of the Lake County Medical Society, Waukegan, Illinois


### A REPORT OF TWO HUNDRED CASES OF TONSILITIS.

*By* J. M. G. CARTER, M. A., M. D., PH. B., President of the Lake County Medical Society, Waukegan, Illinois.

(Read before the Chicago Medical Society, Nov. 16, 1885.)

On the permeable, sandy shore of Lake Michigan, with a prevailing northwest wind for a great part of the year, in connection with the natural humidity of the atmosphere near so large a body of water, we would expect to meet with a large number of cases of throat and chest-diseases. It was under such circumstances that the cases occurred, which I desire to report briefly on this occasion. For the purpose of getting a better understanding, possibly, of the relation existing between tonsilitis, diphtheria, rubeola, rötheln, and scarlatina, it may be well to mention the occurrence of these diseases in the order of their sequence, during the time which is represented by the two hundred cases of tonsilitis that are here reported.

These cases occurred during the last two years. In the fall and early winter of 1883, I had some forty cases of scarlatina. In the months of 1884 the scarlet-fever epidemic subsided, and immediately succeeding it came an epidemic of tonsilitis. Following that, in the summer, we had some cases of rubeola. The measles subsided by fall, and again there were many cases of tonsilitis, and although there were a few cases of mild scarlet-fever through the winter of 1884–85, there were many more of sore-throat. During the spring and summer of 1885 we had an epidemic of rötheln.

During the epidemic, or rather endemic of tonsilitis following the epidemic of scarlet-fever, I treated within the months of March, April and May, about ninety cases of this disease of the throat. During the same months of 1885 I treated sixty cases. The others were somewhat sporadic, and distributed throughout the remaining months of the years 1884 and 1885.

The disease is marked at the beginning by chilliness; aching in the limbs, back of the neck and head; nausea; a feeling of stiffness in the throat. The throat and tonsils are red and the tonsils swollen; and the glands at the angle of the jaw are usually swollen and tender. In 75 per cent. of the cases, on the second day, there are ulcerated patches on the tonsils, and in 5 per cent. a tendency to the formation of a diphtheritic membrane. In 20 per cent. of these cases there are no patches on membranes. The beginning-symptoms are the same in all, and ordinary cases extend over a period of from three to five days,—usually beginning to grow better on the third day. Five cases were sick as long as two weeks; three cases developed into quinsy, and I opened the tonsilar abscess; one case had quinsy and diphtheritic deposits. Two cases had trouble with the glands—one with the sub-maxillary and the other with those of the neck, both on the left side. In both cases it was necessary to lance the abscess. In two cases the swelling of the tonsils was very rapid and the pain of swallowing excruciating. The pulse was usually 120, and temperature 104° in adults. In children, the pulse ranged from 120 to 160, the temperature being the same as in adults. As the swelling of the tonsils began to subside, the fever began to abate. One other case I will mention, because of its close apparent relation to scarlet fever. A young Miss of fourteen was taken with the symptoms described above, the tonsils being very large. There being some scarlet fever about, we were anxious and examined and watched for development of the disease. She recovered in five days, but manifested no other than the symptoms of tonsilitis. Five days later I was called to see one of the children in the same family, and discovered at once a case of scarlet fever. Four of the children had the disease in the next ten days, three of them being malignant cases, of whom two died. Of this family the only child that did not have scarlet fever had a protracted siege of typhoid fever, three months later. Perhaps 25 per cent. of the cases I have reported were left with chronically enlarged tonsils. During the time of the occurrence of these cases, I treated but six cases of diphtheria, and they were not severe. Of the two hundred cases all recovered.

The treatment in these cases varied very little in most cases. It consisted mainly of aconite and belladonna, with, occasionally, chlorate of potassa. Sometimes, especially if there was much disturbance of the stomach, I added gelsemium. This was used internally, usually in small doses, every 30 or

60 minutes. In adults and children old enough to gargle or use a gargle as a throat-bath, I employed local treatment. I have used several gargles—alcohol and water, alcohol and chlorate of potash, water and chlorate of potash, hot water, ice water, salt and vinegar, salt and water, according to circumstances and the condition of the patient. Ordinarily I prefer alcohol and chlorate of potass. where the patient can use a gargle. Excellent external applications are mustard, camphor, kerosene, alcohol, red and black pepper.

In some cases of rapid swelling and intense pain, I secured the quickest results from steam-atomization, using carbolic acid and chlorate of potass. I have used the steam from hot water in many cases with benefit. I was called to a gentleman who was subject to quinsy. He said that he should go through the full course of the disease, he knew, for he had never succeeded in having it checked. I gave him one drop of tincture of aconite and tincture of gelsemium, each, and four drops of tincture of belladonna every hour. I also directed the use of steam, and an alum gargle. The throat grew better, and he recovered without going through any " course " of disease. He has had one similar attack since, and by treating him in the same way we had the same result.

The tincture of the chloride of iron I employed in a few cases. In one case I employed the compound of tincture of iron, carbolic acid, glycerine, chlorate of potash and water, and with no better result than I received from more palatable preparations.

The ordinary course of tonics was prescribed, in some cases, as an after treatment, but generally no tonic treatment was needed. Lactic acid, nitric acid, and hydrochloric acid were freely used in a few cases.

I will report that in the great majority of cases alcohol, chlorate of potass. and water gave the most satisfactory results; while internally tincture of aconite, tincture of belladonna and tincture of gelsemium are equally beneficial.

There must be some cause for such a prevalence of this disease in any locality. There must be a cause for its prevalence at the same season of the year. It will be remembered that the greater number of these cases occurred during the months of March, April and May. In casting about for some special features, which during these months are different from, or in excess of, those of other months, we find an apparent cause in the prevailing northeast wind. During the three months named, the western shore of Lake Michigan has more days of lake-winds than of winds from any other direction. The lake-winds prevail more generally at Milwaukee than at Chicago. During the month of May, for instance, at Milwaukee the easterly winds are to the westerly as 62 to 24, while at Chicago they are as 44 to 40. In this relation, Waukegan more nearly approaches Milwaukee. We usually have an increase of the disease under discussion during the month of July, and during that month there is a preponderance of lake-winds at the points mentioned. At Chicago, the easterly winds are to the westerly as 60 to 33, while at Milwaukee they are as 48 to 37.

At Waukegan, the record for the ozone-test, last year showed that the presence of that agent was very marked during the time when tonsilitis prevailed; that is, the lake-winds seem to sustain some special relation to ozone in the atmosphere. Not having made any experiments with a view to determine this relation, I am not prepared to discuss it. During the last summer, especially in July, northeast and east winds prevailed to a greater extent than usual. Rötheln was the prevailing disease, 75 cases of which occurred in my practice. More than 50 per cent. of these cases were accompanied with sore throat,—the symptoms being precisely the same as in simple tonsilitis. Nearly one-half of the total number of cases of rötheln had ulcerative patches on the tonsils.

Exposure to cold has not been a frequent cause of this sore-throat, for a very large proportion of my cases were parties who had not been exposed to cold; however, such exposure in some cases was known to be the exciting cause. I would mention one other point. I have noticed that rheumatic and nervous patients were not so well during these periods.

From a consideration of all these points, I have been led to suspect that the disease under discussion, like nervous disorders and rheumatism, is affected by, if not due to, the electrical condition of the atmosphere; but whether to an excess or a deficiency of electricity I have not sufficient data to determine accurately. We know this: that the disease prevails when there is a humid atmosphere, an excess of ozone, a lake-wind, and a low temperature. We know, further, that these agencies work changes in the electrical condition of the atmosphere.

